# Cavitating flow-induced unsteady pressure pulsations in a low specific speed centrifugal pump

**DOI:** 10.1098/rsos.180408

**Published:** 2018-07-04

**Authors:** Ning Zhang, Bo Gao, Zhong Li, Qifeng Jiang

**Affiliations:** 1 School of Energy and Power Engineering, Jiangsu University, Zhenjiang 212013, People's Republic of China; 2 Key Laboratory of Fluid and Power Machinery, Ministry of Education, Xihua University, Chengdu 610039, People's Republic of China

**Keywords:** centrifugal pump, cavitating flow, pressure pulsation, blade-passing frequency, root mean square value

## Abstract

With the development of cavitation, the high-energy pressure wave from a cavitation bubble collapsing is detrimental to the stable operation of centrifugal pumps. The present paper concentrates on pressure pulsations under cavitation conditions, and pressure amplitudes at the blade-passing frequency (*f*_BPF_) and RMS values in the 0–500 Hz frequency band are combined to investigate cavitation-induced pressure pulsations. The results show that components at *f*_BPF_ always dominate the pressure spectrum even at the full cavitation stage. For points P1–P7 on the volute side wall, with a decreasing cavitation number, the pressure energy first remains unchanged and then it rises rapidly after the critical point. For point In1 in a volute suction pipe located close to the cavitation region, the pressure energy changes slightly at high cavitation numbers; then for a particular cavitation number range, the pressure energy decreases, and finally increases again. For different flow rates, the pressure energy at the critical point is much lower than the initial amplitude at the non-cavitation condition for In1. This demonstrates that the cavitation cloud in the typical stage is partially compressible, and the emitted pressure wave from a collapsing cavitation bubble is absorbed and attenuated significantly. Finally, this leads to the pressure energy decreasing rapidly for the measuring point In1 near the cavitation region.

## 1. Introduction

For centrifugal pumps, cavitation usually occurs in the blade channels, even at the volute tongue region, due to a drop in the inlet suction pressure [1,2]. Owing to the blockage effect of cavitation bubbles on the blade channels, the performance of the centrifugal pump can be significantly affected, and this is usually known as the sudden head drop [3]. In addition, cavitation erosion on the blade may occur as a result of the bubbles' collapsing effect [4]. Along with cavitation development, cavitation-induced high-energy pressure pulsation, vibration and noise can be severe and can also cause unexpected results that are detrimental to the stable and safe operating of the pumping system [5]. Consequently, it is essential to investigate the related flow phenomena caused by cavitation and to find an effective way to detect and avoid cavitation during pump operation.

During cavitation bubble bursting, a shock wave can be generated in an extremely short time for a single bubble, usually of the order of micro-seconds. For a cavitation cloud, relatively low-frequency signals are excited. Investigations are often focused upon the evolution of a cavitation cloud. Ji *et al.* [6–8] numerically investigated the evolution of cavitation cloud shedding from a hydrofoil and attempted to clarify the shedding characteristics from the cavitation–vortex interaction aspect. For cavitation-induced pressure pulsation characteristics, it is proved that pressure amplitude is closely related to the volumetric change of the cavitation cloud [9]. In the field of hydraulic turbines, some investigations focus on cavitation-induced pressure pulsation. Rus *et al.* [10] investigated the relationship between the cavitation structure and pressure pulsation of a Kaplan turbine and revealed preliminary findings that pressure energy varies with cavitation development. For centrifugal pumps, cavitation can also have an effect on the pressure pulsation characteristics. Recently, Lu *et al.* [11] investigated unsteady cavitation characteristics in a centrifugal pump, and pressure pulsations were extracted by placing a pressure transducer at the pump inlet suction. The relations between the typical frequency and unsteady cavitation were investigated. Wang *et al.* [12] predicted cavitating flow structures in an impeller, and cavitation distributions and blade loads at different stages were discussed.

To detect cavitation, the most commonly used method is the determination of the pump head drop, and the 3% head drop criterion is applied to determine cavitation occurrence [13,14]. However, this is not an accurate method, as cavitation usually occurs much earlier than the 3% head drop. In other words, the internal flow structures, pressure pulsation, vibration and noise are affected before the 3% head drop point, as proved by many researchers [15–17]. Because cavitation-induced noise and vibration are known to occur, some researchers have used the emitted noise and the induced vibration to detect cavitation [18,19]. Čudina *et al.* [20–22] investigated the noise characteristics of a centrifugal pump under cavitating flow conditions, and found that cavitation significantly affected discrete frequencies in the noise spectrum. The varying trend of noise energy versus cavitation number was established and discussed. Zhang *et al*. [23] investigated the vibration characteristics caused by the cavitation of a centrifugal pump with a slope volute. Vibration signals were divided into four frequency bands to discuss the influence of cavitation on vibration energy [23].

In this work, pressure pulsation characteristics induced by cavitating flow are analysed. Pressure signals were measured using high-frequency response pressure transducers mounted on the volute casing. This work attempts to illustrate the influence of cavitation on the pressure spectrum both for the discrete component and for the pressure energy in a particular frequency band. Finally, investigation of the pressure pulsation characteristics versus different cavitation stages is carried out, and the influence of cavitation on pressure pulsations in centrifugal pumps is revealed.

## 2. Experimental set-up

In this paper, a low specific speed (*n*_s_ = 69) centrifugal pump is used to investigate the influence of cavitation on pressure pulsation. The main design parameters of the model pump are presented in table 1. The blade of the impeller is designed to a two-dimensional shape and the shapes of the volute cross sections are rectangular.

**Table 1.** Main design parameters of the model pump.

parameters	value
nominal flow rate *Q*_d_	55 m^3^ h^−1^
nominal head *H*_d_	20 m
nominal rotating speed *n*_d_	1450 r min^−1^
specific speed $n_{s}=3.65n_{d}\sqrt{Q_{d}}/{H_{d}}^{0.75}$	69
blade number *Z*	6
impeller inlet diameter *D*_1_	80 mm
impeller outlet diameter *D*_2_	260 mm
impeller outlet width *b*_2_	17 mm
volute inlet diameter *D*_3_	290 mm
volute outlet diameter *D*_4_	80 mm
blade outlet angle *β*_2_	30°
wrap angle *ϕ*	115°
angle of volute tongue *α*	20°
tangential velocity at the trailing edge of impeller blade *u*_2_	19.7 m s^−1^
impeller rotating frequency *f*_n_	24.2 Hz
blade-passing frequency *f*_BPF_	145 Hz

**Figure 1.**
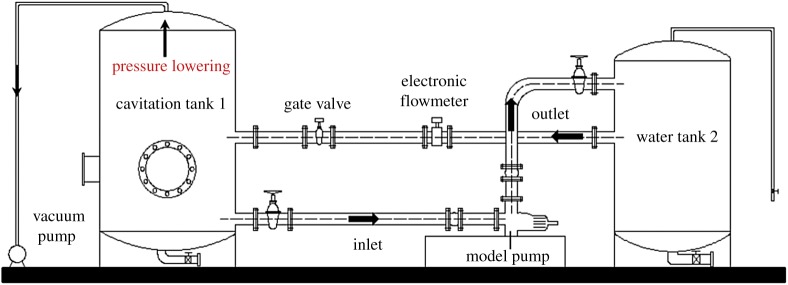
Closed test platform.

Cavitating flow-induced pressure pulsation experiments are carried out on a closed test platform as shown in figure 1. Pressure gauges, with an uncertainty of ±0.1%, are placed at the pump inlet and outlet suctions to measure the pressure value of the model pump and to obtain the pump head. To make the pump work under the cavitation conditions, a vacuum pump is used to extract air from cavitation tank 1. Consequently, the pressure in the experimental system is reduced gradually. An electronic flowmeter with a measuring accuracy of ±0.2% is applied to obtain the flow rate of the model pump. During the experiments, the gate valve at the pump inlet is fully opened, so cavitation cannot occur in the valve. To make the pump work under different conditions, the gate valve at the outlet pipe is adapted to adjust the flow rate. Furthermore, a frequency inverter is used to guarantee that the pump is working at the rated speed, namely 1450 r min^−1^.

**Figure 2.**
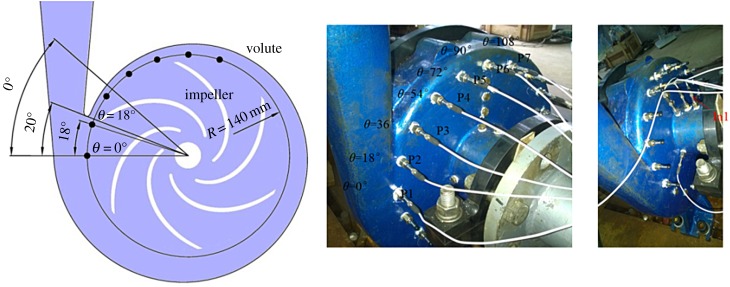
Positions of the pressure transducers.

Seven threaded holes located between the impeller outlet and the volute inlet area were drilled in the volute side wall to mount the high-frequency response pressure transducers, as shown in figure 2. The angle of the adjacent holes was 18°. Pressure transducers were mounted on the volute casing through the threaded holes. In addition, at the volute suction pipe, another pressure transducer was mounted and this was designated as sensor In1. Cavitation usually occurs at the blade leading edge, so sensor In1 was closer to the cavitation region than sensors P1–P7. The pressure transducers that we used, i.e. the PCB113B27 series, have a high measuring accuracy, with error rates lower than 0.2%. The angle of the point at the eighth cross section of the volute casing, namely point P1, is defined as 0°. Going in a clockwise direction, the angle increases by increments of 18°. For the cavitation cloud in pumps, the emitted frequency signals are much lower than the noise from a single cavitation bubble collapse. Hence, during cavitation experiments, the adopted bandwidth of the pressure spectrum was set to 10 240 Hz, with a corresponding sampling frequency of 20 480 Hz. To have a high resolution of the pressure spectrum, the frequency resolution was defined as 0.5 Hz. During signal processing, the Hanning window was used for the fast Fourier transform (FFT).

## 3. Results and discussions

### 3.1. Cavitation performance of the model pump

In centrifugal pumps, to indicate the occurrence of cavitation, the net positive suction head (NPSH) is usually used as defined in equation (3.1). The 3% head drop point is used to determine the full cavitation condition, and it is commonly defined as the critical net positive suction head (NPSH_c_). The determination method is known as the 3% head drop criterion,

3.1 $$\text{NPSH}=\frac{P_{\mathrm{in}}-P_{v}}{\rho g}+\frac{v_{\mathrm{in}}^{2}}{2g},$$

where *v*_in_ is the velocity at the pump inlet, *P_v_* is the local saturated vapour pressure and *P*_in_ is the absolute static pressure at the pump inlet.

For dimensionless treatment of NPSH, the cavitation number, also called the Thoma number, is defined as

3.2 $$\sigma =\frac{\text{NPSH}}{H},$$

where *H* is the measured total delivery pump head at a nominal flow rate.

**Figure 3.**
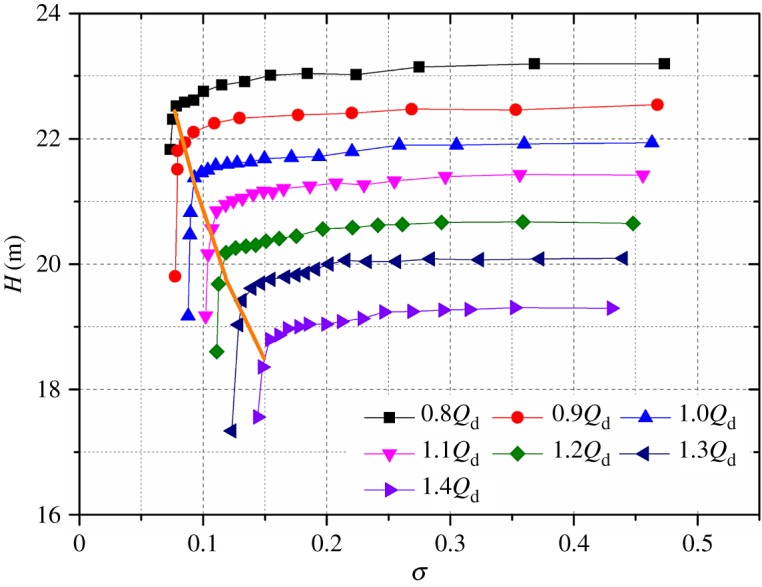
Cavitation performance of the model pump.

Cavitations at flow rates of 0.8*Q*_d_ to 1.4*Q*_d_ are investigated in the present work. Cavitation performances of the model pump are presented in figure 3. It is clear that the pump performance is almost unaffected at high cavitation numbers. After the 3% head drop point, it decreases suddenly. From the 3% head drop criterion, the critical NPSHs of the model pump at flow rates of 0.8*Q*_d_–1.4*Q*_d_ are 1.68 m, 1.74 m, 2.0 m, 2.4 m, 2.55 m, 2.95 m, 3.3 m, respectively, and the corresponding cavitation numbers are 0.076, 0.079, 0.091, 0.109, 0.116, 0.134, 0.150.

### 3.2. Cavitation-induced pressure pulsations

A pressure coefficient is used for dimensionless treatment of the pressure amplitude at a typical frequency and is defined as

3.3 $$c_{p}=\frac{A}{0.5\rho u_{2}^{2}},$$

where *ρ* is the water density, *u*_2_ is the tangential velocity at the trailing edge of the impeller blade and *A* is the amplitude of pressure pulsation.

**Figure 4.**
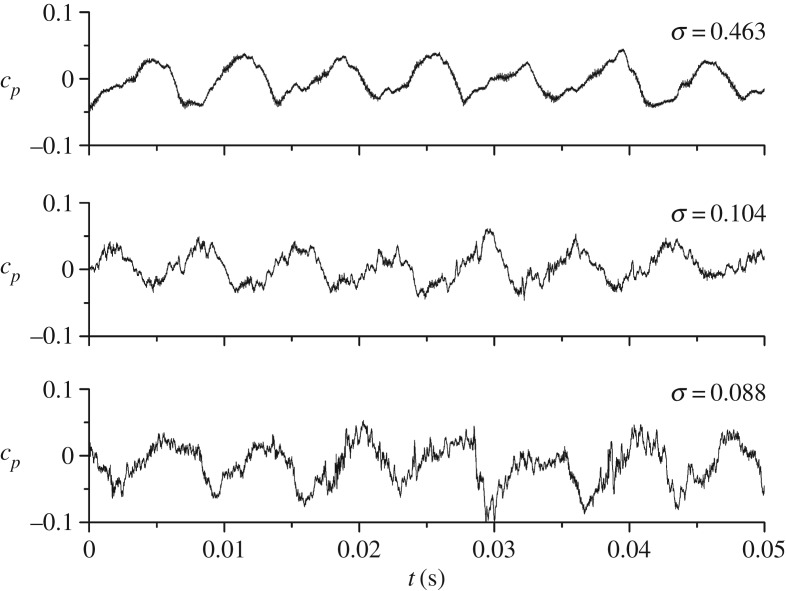
Time-domain pressure signals at different cavitation numbers.

Figure 4 first presents time-domain pressure signals of P3 at different cavitation numbers under a nominal flow rate. As observed at a high cavitation number *σ* = 0.463, pressure pulsation signals show quasi-periodic characteristics, and within a rotating cycle *t* = 0.0414 s, six peaks and valleys occur due to rotor–stator interaction between the impeller and the volute tongue [24]. When the cavitation number decreases to *σ* = 0.104, i.e. higher than the critical point, the pressure signals are affected by the occurrence of cavitation bubbles. The smooth pressure pulsation signals are influenced, as characterized by the evident burr-shaped spikes developing. However, at this stage, the pressure pulsation amplitude remains almost unchanged. When cavitation decreases lower than the critical point, namely the 3% head drop point, the full cavitation status within the impeller channel is considered to develop at *σ* = 0.088. Under these conditions, a large-scale cavitation cloud exists at the blade inlet region. The pressure amplitude increases significantly due to cavitation bubble collapse and the volumetric change of the cavitation cloud compared with the non-cavitation condition [25].

**Figure 5.**
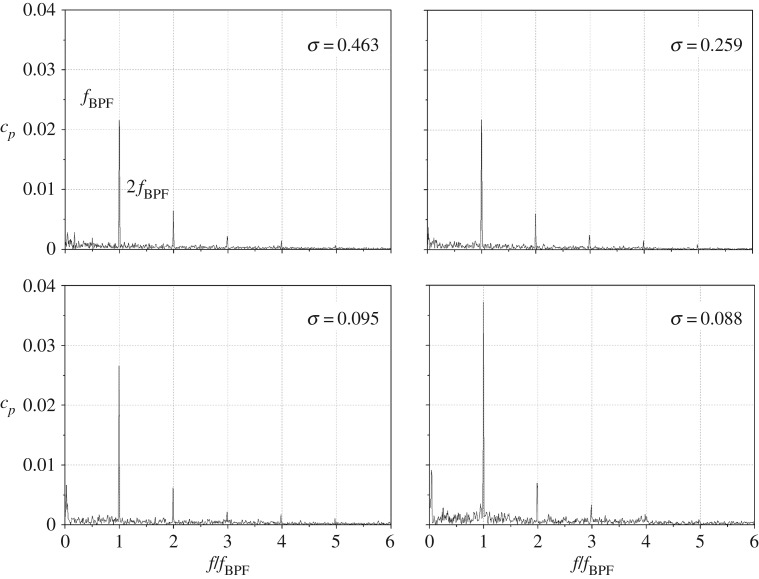
Pressure spectra of P3 at various cavitation numbers under nominal flow rate.

Pressure spectra of P3 at different cavitation numbers at the nominal flow rate after frequency transformation of the time-domain pressure signals are presented in figure 5. Owing to cavitation bubble collapse and the volumetric change of the cavitation cloud, the pressure spectrum would be affected obviously. In the non-cavitation condition *σ* = 0.463, evident components at the blade-passing frequency *f*_BPF_ and the harmonics 2*f*_BPF_, 3*f*_BPF_ are captured in the pressure spectrum. The amplitude at *f*_BPF_ is much larger than that at other frequencies, so it maintains a predominant role in the pressure spectrum in the non-cavitation condition. With the cavitation number decreasing to *σ* = 0.259, the pressure spectrum is similar to that at *σ* = 0.463, and the pressure amplitude changes little. When the cavitation number decreases further to *σ* = 0.095 around the critical point, the pressure spectrum is affected obviously. As observed, the amplitude at *f*_BPF_ rises. For the full cavitation status at *σ* = 0.088, the amplitude at *f*_BPF_ is nearly 1.7 times that in the non-cavitation condition. Commonly, it is considered that, for single cavitation bubble bursting, high-frequency noise of greater than 10 kHz would be emitted [1]. During the experiments, the pressure transducers were located some distance from the cavitation-developing region, and this may have a significant effect on the high-frequency signal detected. Owing to the rapid degradation of the emitted shock wave energy, high-frequency signals of greater than 1000 Hz in the pressure spectrum were not captured in the centrifugal pumps during the experiments. Hence, in the present work, emphasis is laid upon the frequencies within 1000 Hz to illustrate the correlation between cavitation developing and the pressure pulsation characteristics.

**Figure 6.**
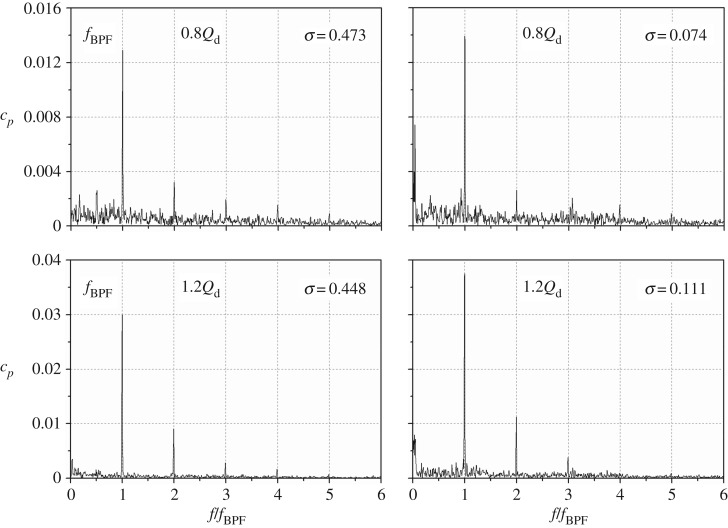
Pressure spectra of P3 in the non-cavitation and full cavitation conditions at flow rates of 0.8*Q*_d_ and 1.2*Q*_d._

To clarify the influence of the pump working conditions on the pressure spectrum at the cavitation condition, figure 6 shows the pressure spectra in the non-cavitation and full cavitation conditions for flow rates of 0.8*Q*_d_ and 1.2*Q*_d_. When the pump works at a partial flow rate, the pressure spectrum is similar to that at the nominal flow rate. It is observed that, at 0.8*Q*_d_, the pressure amplitude at the blade-passing frequency under full cavitation status *σ* = 0.074 has an increment of 7% compared with that in the non-cavitation condition *σ* = 0.473. At 1.2*Q*_d_, the increment is about 25% at *σ* = 0.111 in comparison with that at *σ* = 0.448. From the above analysis, it can be concluded that cavitation surely affects the component at *f*_BPF_ for all the concerned flow rates.

**Figure 7.**
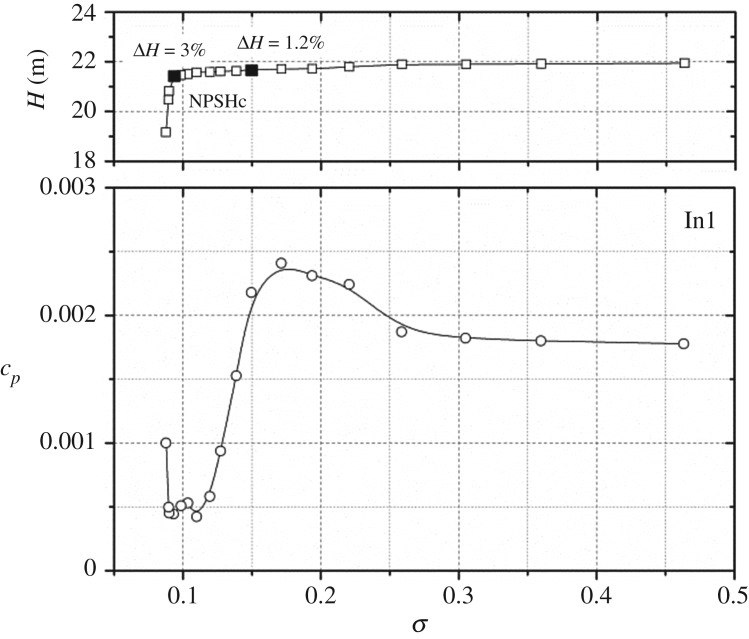
Pressure amplitude at *f*_BPF_ versus cavitation number under nominal flow rate at point In1.

With the development of cavitation, the cavitation structure and cavitation region would obviously change. Hence to clarify the influence of the cavitation-developing process on the pressure amplitude at *f*_BPF_ under nominal flow rate, figure 7 presents pressure amplitudes at *f*_BPF_ versus the cavitation number at point In1. As observed at high cavitation number *σ* > 0.305, the pressure value at the blade leading edge is much larger than the local saturated vapour pressure, and consequently no cavitation bubble occurs in the impeller. At this stage, pressure amplitudes are little affected and remain nearly unchanged. With the decrease in cavitation number from *σ* = 0.305 to *σ* = 0.15, the pressure amplitude rises for point In1. The corresponding head drop is about 1.2%. This means that, at this stage, cavitation bubbles occur within the impeller channels, and the blade channels are slightly blocked by the cavitation cloud. With cavitation decreasing to *σ* = 0.104, namely the point a little higher than the critical point, the pressure amplitude decreases significantly, and the amplitude is much lower than that in the non-cavitation condition. For point In1, the pressure amplitude at *σ* = 0.104 is about 30% of that at *σ* = 0.463. When the cavitation number decreases further to lower than the critical point, full cavitation is considered to develop in the impeller channels. At this stage, the pressure amplitude starts to rise again. So it is inferred that the varying trend of pressure amplitude is related to the corresponding cavitation status in the model pump.

**Figure 8.**
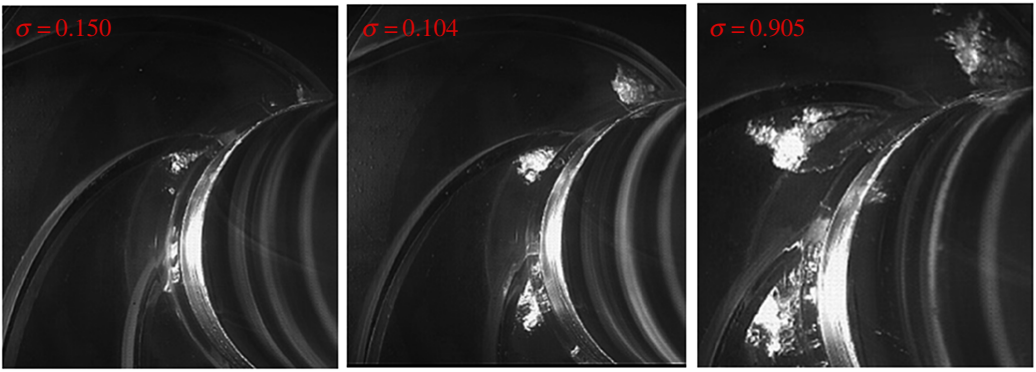
Cavitation structures at different cavitation numbers.

The corresponding cavitation flow structures at different cavitation numbers are presented in figure 8. A detailed description of the cavitation visualization experiment can be found in our previous work for the model pump [26]. It is observed that small cavitation bubbles occur at the blade suction side near the leading edge at cavitation number *σ* = 0.150. At this stage, the corresponding head drop in figure 3 is about 1.2%. So, from the head drop, it is usually considered that cavitation does not develop in the impeller channels. However, from cavitation visualization, it is apparent that cavitation bubbles are captured long before the normal 3% head drop criterion. Owing to the development of cavitation bubbles in the impeller, pressure pulsation is affected at this stage. With the decrease in the cavitation number to *σ* = 0.104, a little higher than the critical cavitation number, the cavitation region in the blade channel expands, which occupies a larger area of the blade inlet. At this condition, the corresponding head drop is about 2%. From figure 7, it is noted that the pressure pulsation amplitude reaches a minimum point at *σ* = 0.104. When the cavitation number decreases below the critical point at *σ* = 0.905, it is observed that almost half of the blade inlet area is occupied by cavitation bubbles. At this stage, both the performance and pressure pulsation energy are affected significantly.

**Figure 9.**
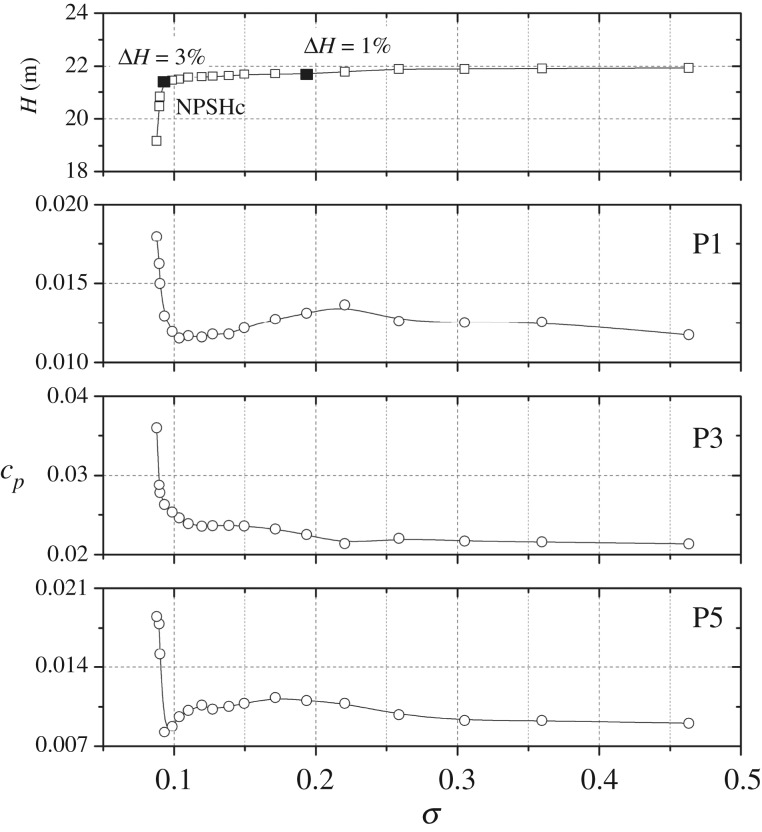
Pressure amplitudes at *f*_BPF_ versus cavitation number under nominal flow rate for points P1, P3, P5.

To clarify the influence of the cavitation-developing process on pressure pulsation characteristics of the points on the volute side wall, figure 9 shows pressure amplitudes at *f*_BPF_ of points P1, P3 and P5. It can be observed that all of the varying trends are similar to those at point In1; however, some differences exist. At a high cavitation number, for the three measuring points, pressure amplitudes remain almost unchanged. From cavitation number *σ* = 0.305 to *σ* = 0.191, pressure amplitudes increase, but the increment is not significant. From cavitation number *σ* = 0.191 to *σ* = 0.104, pressure amplitudes at P1 and P5 start to decrease. At *σ* = 0.104, pressure amplitudes for the three points are nearly identical to the corresponding initial pressure amplitudes in the non-cavitation condition. However, for point In1, the pressure amplitude at this cavitation number is much lower than the initial value as shown in figure 7. When the cavitation number drops below the critical point *σ* = 0.091, pressure amplitudes rise rapidly again. Especially at the full cavitation status *σ* = 0.088, pressure amplitudes are much higher than the initial values.

The varying trends of pressure amplitudes are associated with the corresponding cavitation stage within the model pump. At high cavitation number, no cavitation bubbles occur in the pump, so pressure amplitudes are not affected and remain unchanged. From cavitation number *σ* = 0.305 to *σ* = 0.191, a small cavitation cloud develops, and the internal flow structure is affected by the occurrence of cavitation, leading to the pressure amplitude changing slightly. From *σ* = 0.150 to *σ* = 0.104, for the measuring points concerned, the pressure amplitudes exhibit a steeply decreasing trend. The decreases are related to the cavitation status. It is thought that, at this stage, the cavitation clouds are partially compressible. The emitted energy from the cavitation bubble collapse is absorbed and weakened by the compressible cavitation structure, and in previously published work [27], the cavitation status is considered to be bubble cavitation. Finally, within this cavitation number range, pressure amplitudes are attenuated significantly. For point In1 near the cavitation region, the compressible effect of the cavitation structure on the pressure amplitude is more significant. As noted, the pressure amplitude at the critical point is even lower than the initial amplitude in the non-cavitation condition due to the absorption of the pressure pulsation energy of the cavitation cloud. For points P1, P3, P5 far away from the cavitation region, the influence is weakened and pressure amplitudes only show a slightly decreasing trend. With the cavitation number lower than the critical point, the cavitation cloud becomes incompressible. From our previous work [26], it is found that the cavitating flow structure is significantly different from the non-full cavitation stage. At this cavitation number, the cavitation region can be divided into two separate parts, namely, the two-phase flow region and the vapour flow region. A free surface develops between the two-phase flow and the vapour flow; it also exists between the vapour flow and the main flow. Owing to the existence of interfaces between two-phase flows, the cavitation status changes from bubble cavitation to an incompressible cavitation stage. Thus, non-bubble cavitation cannot absorb the pressure pulsation energy emitting from cavitation bubble collapse. Finally, at this cavitation stage, the pressure amplitudes rise rapidly again. Such a varying trend agrees well with the previous works [10,17].

**Figure 10.**
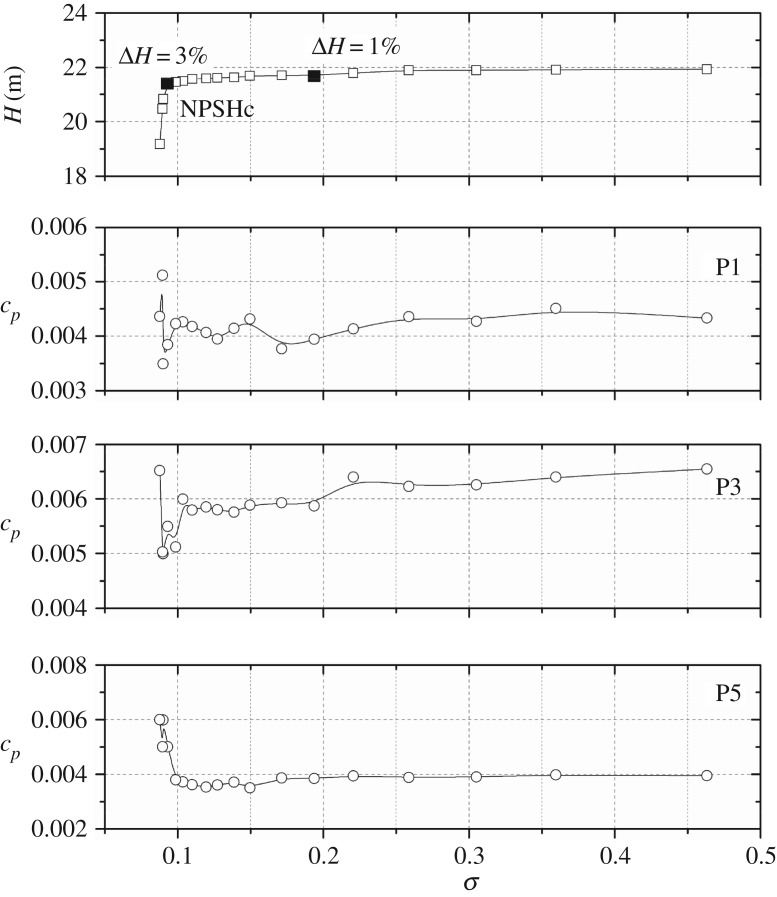
Pressure amplitudes at 2*f*_BPF_ versus the cavitation number under nominal flow rate at points P1, P3, P5.

From figures 5 and 6, it is observed that, in the pressure spectrum, the harmonic of the blade-passing frequency at 2*f*_BPF_ occurs at various flow rates and cavitation numbers. Hence to clarify the influence of the cavitation-developing process on pressure amplitudes at 2*f*_BPF_, figure 10 shows pressure amplitudes versus the cavitation number at points P1, P3 and P5. As observed, the varying trends are similar to those at *f*_BPF_. At high cavitation numbers, pressure amplitudes remain almost unchanged. For points P1 and P3, from cavitation number *σ* = 0.104 to *σ* = 0.091, the pressure amplitude decreases, and after that it rises rapidly. For point P5, when the cavitation number is lower than the critical point, the pressure amplitude increases steeply. However, the decreasing trend is not observed. It can be concluded that, for components at 2*f*_BPF_, pressure amplitudes are affected when the cavitation number decreases to about the critical point.

**Figure 11.**
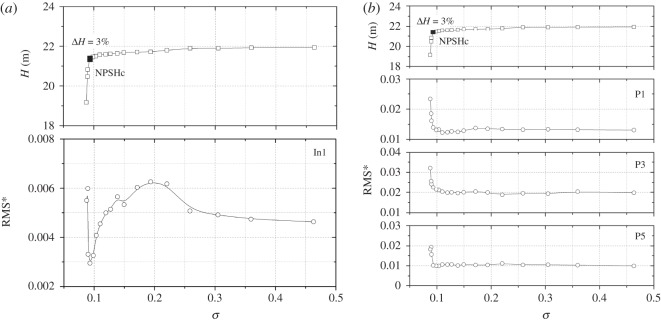
RMS* values in the 0–500 Hz frequency band versus cavitation number at a nominal flow rate: (*a*) point In1, (*b*) points P1, P3, P5.

To evaluate the pressure energy in a particular frequency band considering the components at each frequency, the RMS method is introduced as defined in equation (3.4). For non-dimensionless treatment, the RMS is then converted to RMS* using the formula in equation (3.5). In the pressure spectrum, it is found that, at high frequency (*f* > 500 Hz), no evident peaks can be detected. Hence to clarify the effect of cavitation on the total pressure energy, figure 11 shows RMS* values in the 0–500 Hz frequency band at points In1, P1, P3 and P5.

3.4 $$\text{RMS}=\frac{1.63}{2}\sqrt{\frac{1}{2}\left(\frac{1}{2}A_{0}^{2}+\sum_{n=2}^{n-1}A_{n-1}^{2}+\frac{1}{2}A_{n}^{2}\right)},$$

where *A_n_* represents peaks at different frequencies. For non-dimensionless treatment, RMS is converted to RMS*,

3.5 $$\text{RM}{\text{S}}^{*}=\frac{\text{RMS}}{\rho u_{2}^{2}}.$$

It is observed that the varying trends of different points are not identical. For point In1 located at the volute inlet suction, the pressure amplitude also shows unchanged characteristics at high cavitation numbers larger than *σ* = 0.3; after that it rises. When the cavitation number decreases to less than *σ* = 0.150, the pressure amplitude decreases rapidly; finally, it increases again. The varying trend is associated with the cavitation status as discussed in figure 8. For points P1, P3 and P5, the rapidly decreasing phenomenon is not observed. It is noted that when the cavitation number is larger than the critical point, pressure amplitudes remain nearly unchanged. The pressure amplitude rises rapidly when the cavitation number decreases below the critical point. The varying trends are similar to those in figure 9.

**Figure 12.**
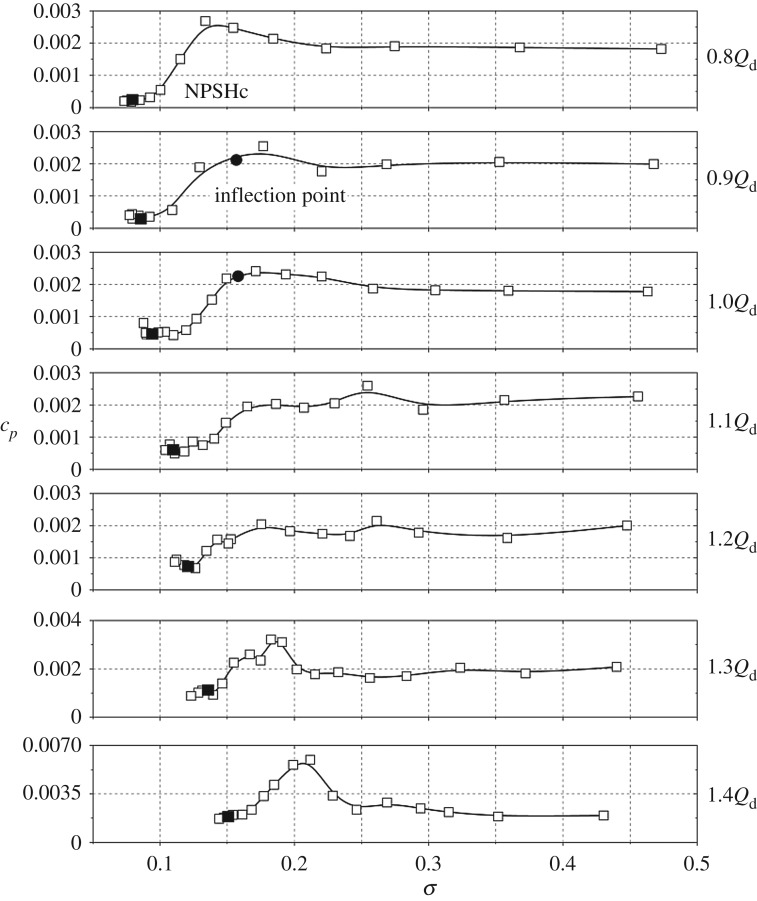
Pressure amplitudes at *f*_BPF_ of point In1 versus cavitation number under various flow rates.

For centrifugal pumps, flow structures within the impeller and the volute are closely associated with the operating condition, and this means that the flow rate often has a significant influence on the internal flow characteristics. So it is essential to clarify the effect of flow rate on the pressure amplitude in the cavitation condition. Figure 12 presents pressure amplitudes at *f*_BPF_ versus cavitation number for flow rates from 0.8*Q*_d_ to 1.4*Q*_d_ at point In1.

From figure 12, it is noted that the varying trends at different flow rates are similar. For flow rates from 0.8*Q*_d_ to 1.2*Q*_d_, at high cavitation number, pressure amplitudes change little. When the cavitation number decreases to a typical inflection point, as seen by the dots in the figure, pressure amplitudes start to decrease rapidly. For all the concerned flow rates, pressure amplitudes at the critical point NPSH_c_ are much smaller than the initial pressure amplitudes in the non-cavitation condition due to the partially compressible cavitation cloud. For high flow rates at 1.3*Q*_d_ and 1.4*Q*_d_, some differences are observed compared with low flow rates. It is noted that, for 1.4*Q*_d_, before the inflection point, the pressure amplitude exhibits an increasing trend from cavitation number *σ* = 0.246 to *σ* = 0.199. The pressure amplitude is nearly three times the initial amplitude. For 1.3*Q*_d_, the pressure amplitude has an increment of 54% compared with the initial amplitude. At extremely high flow rates, cavitation develops much faster than at low flow rates [16]. So at the inflection point, the scale and region of the cavitation cloud are large enough to affect pressure amplitudes. The effect of cavitation on the internal flow structure could be the reason for pressure amplitudes rising before the inflection point at high flow rates.

**Figure 13.**
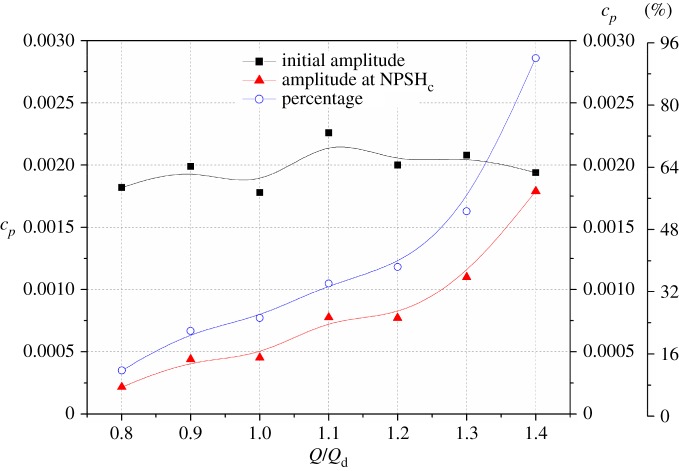
Comparison of amplitudes at *f*_BPF_ for In1 under non-cavitation and critical point NPSH_c_ conditions.

As observed in figure 12, pressure amplitudes at the critical point are smaller than those in the non-cavitation condition. To make a comparison, figure 13 shows the initial amplitudes and amplitudes at NPSH_c_ under various flow rates. The blue curve represents the percentage of amplitudes at NPSH_c_ compared with the initial amplitudes. For initial amplitudes at different flow rates, the values show a small discrepancy, but are nearly identical. For amplitudes at the critical point, it is evident that the pressure amplitude increases significantly versus flow rate. Especially at 1.4*Q*_d_, the amplitude at the critical point is very close to the initial value. From the percentage value, it is noted that, at a low flow rate of 0.8*Q*_d_, the amplitude at the critical point just reaches 12% of the initial value. With the flow rate increasing, the percentage rises rapidly, and at 1.4*Q*_d_ it reaches 92%. From the comparison, it can be concluded that the compressible effect of the cavitation cloud has a greater effect on pressure amplitudes at low flow rates, and it leads to the pressure amplitude decreasing rapidly. With an increase in flow rate, the influence is attenuated as seen at 1.4*Q*_d_.

**Figure 14.**
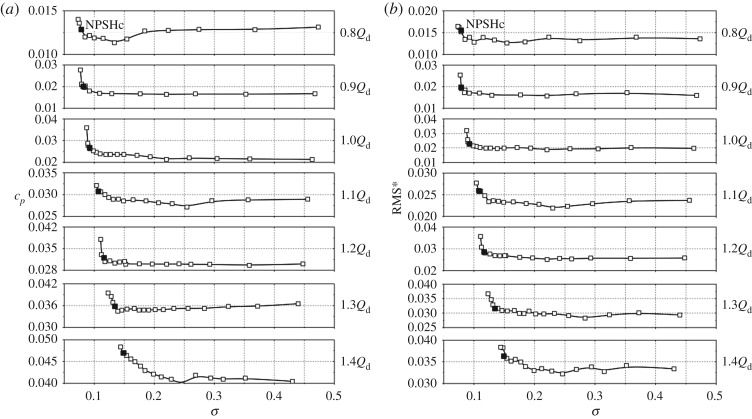
Pressure amplitudes of point P3 versus cavitation number under various flow rates: (*a*) pressure amplitudes at *f*_BPF_, (*b*) RMS* values in the 0–500 Hz frequency band.

Figure 14 presents pressure amplitudes at point P3 versus cavitation number when the pump works at various flow rates. As observed, the varying trends of pressure amplitudes at *f*_BPF_ and RMS* values in the 0–500 Hz frequency band are similar. For different flow rates, pressure amplitudes first remian unchanged, and after the critical point pressure amplitudes rise rapidly. However, from comparison with point In1, it is found that the decreasing trend of the pressure amplitude in any particular cavitation number range does not occur. The reason is related to the far distance of the point P3 from the cavitation-developing region, so the compressible effect of the cavitation cloud is not significant.

From the above analysis, it is concluded that the influence of flow rate on pressure amplitude under cavitation conditions is consistent. For point In1 near the cavitation region, pressure amplitudes are more easily affected by cavitation structure. Within the typical cavitation number range, pressure amplitudes are attenuated by the compressible effect of the cavitation cloud. For point P3, such a phenomenon is not observed. This means that the distance of the measuring point from the cavitation region would have an evident influence on the pressure amplitude.

**Figure 15.**
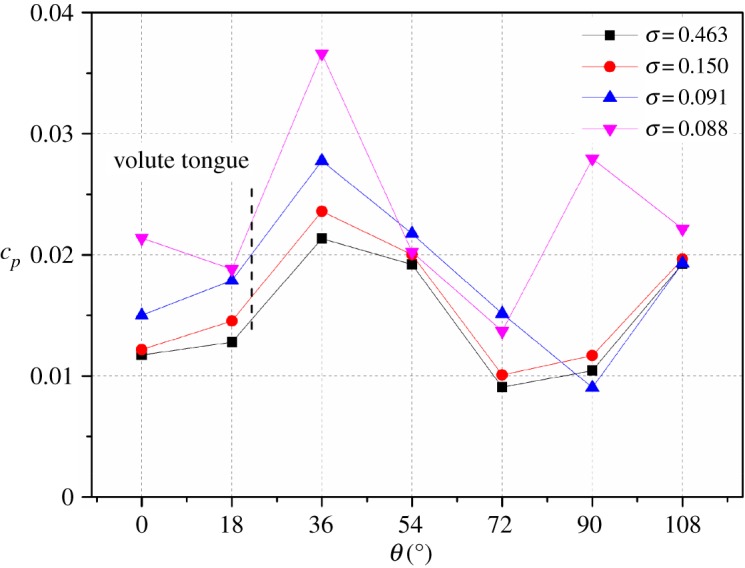
Angular distributions of pressure amplitudes at *f*_BPF_ for different cavitation numbers at nominal flow rate.

During experiments, seven pressure transducers are mounted on the periphery of the volute side wall. To investigate the influence of cavitation at these seven points, figure 15 presents the angular distributions of amplitudes at *f*_BPF_ under four cavitation numbers when the model pump works at a nominal flow rate. For non-cavitation conditions *σ* = 0.463 and *σ* = 0.150, peaks and valleys can be seen from the varying curve, and this is caused by the rotor–stator interaction between the impeller and volute tongue. The influence of cavitation is not obvious, and pressure amplitudes at two cavitation numbers are nearly identical. The maximum values occur at *θ* = 36°, namely the region after the volute tongue, and the reason for this is the corresponding flow structures in this region. A detailed analysis can be found in our previous research work [28]. When the cavitation number decreases to the critical point *σ* = 0.091, the varying trend is the same as that in the non-cavitation condition. Overall, pressure amplitudes start to rise, especially at an angle of *θ* = 36°. The increment reaches 30% compared with that at *σ* = 0.463. Furthermore, when the cavitation number drops to *σ* = 0.088, for all the concerned measuring points, pressure amplitudes increase obviously. For the critical point, the average increment of pressure amplitudes at the seven points is 24%, and it reaches 63% at cavitation number *σ* = 0.088.

### 3.3. Visualization of cavitating flow

The influence of cavitation on pressure pulsation is closely associated with the corresponding cavitating flow structures within the model pump, and we expect to reveal the changing characteristics of the pressure pulsation versus cavitation number from cavitation flow structures.

**Figure 16.**
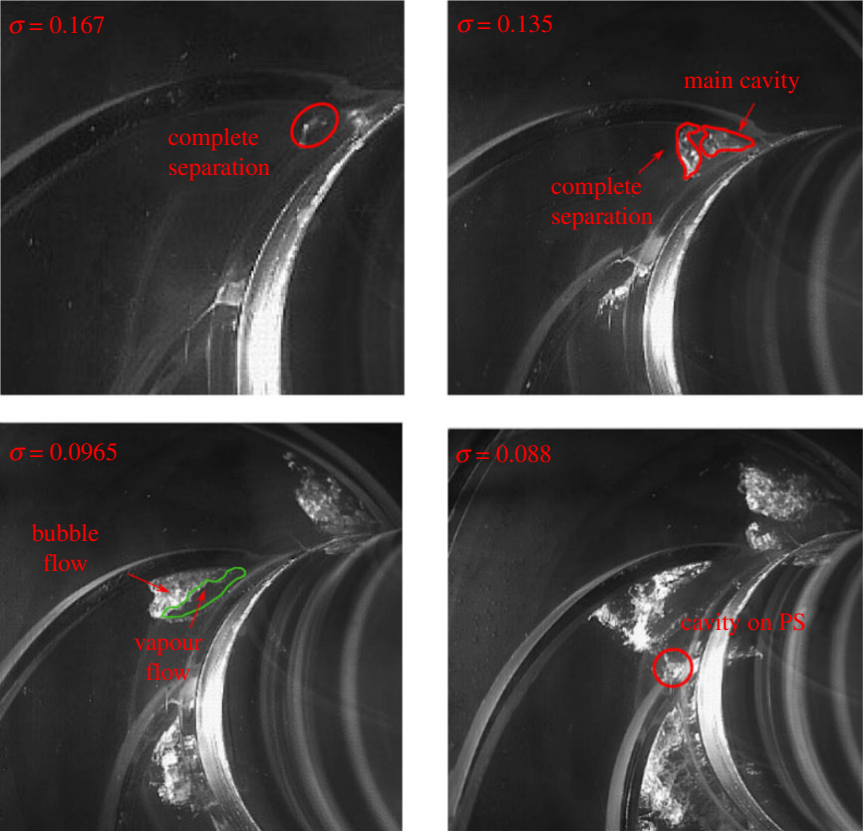
Cavitating flow structures at different cavitation numbers under nominal flow rate.

Figure 16 presents cavitating flow structures at different cavitation numbers at a nominal flow rate. It is very difficult to capture the small cavitation structure at a large cavitation number due to the limitation of the test platform. So emphasis is laid upon cavitation at small cavitation numbers. It is noted that, at cavitation number *σ* = 0.167, cavitation is formed on the blade suction side near the leading edge. A part of the cavitation cloud separates from the main cavity. With the impeller rotating, the cavitation cloud can move with the main flow to the blade downstream; finally, it collapses in the high pressure region with an intense implosion. At *σ* = 0.135, the cavitation region expands, and it occupies a larger blade channel area. In addition, the separated cavitation cloud can also be observed; it would also experience the collapsing process and emit a huge amount of energy. With the cavitation deceasing to *σ* = 0.0965 around the critical point, the cavitation structure is significantly different from that at high cavitation numbers. It is evident that the cavitation structure could be divided into two separate parts, namely the vapour flow region and the two-phase flow region (usually called bubble flow). When the cavitation number decreases to *σ* = 0.088, lower than the critical point, the cavitation structure expands and even develops on the blade pressure side. At this stage, a large area of the blade leading region is occupied by cavitation bubbles, so the corresponding pump head drops rapidly.

As presented in figure 7 for point In1 close to the cavitation region, the pressure amplitude shows a decreasing trend at cavitation numbers of *σ* = 0.15 to *σ* = 0.104; then the pressure amplitude increases from cavitation number *σ* = 0.104 to full cavitation *σ* = 0.088. The typical changing characteristics are closely related to the corresponding cavitation structures. It is considered that, at *σ* = 0.135, the cavitation structure is in two-phase flow, thus the cavitation structure is compressible. The emitted energy from the cavitation cloud collapse can be absorbed and attenuated due to the compressible cavitation structure. This is the reason why the pressure amplitude decreases with cavitation numbers from *σ* = 0.15 to *σ* = 0.104. For the cavitation flow structure at the near-critical point *σ* = 0.0965, significant vapour flow is developed. The cavitation structure at this condition is incompressible due to the occurrence of vapour flow. Finally, the emitted energy from cavitation bubble collapse could not be reduced, leading to an increase in the pressure amplitude. The changing status of the cavitation structure is the reason why the pressure amplitude first decreases and then rises at low cavitation numbers. Such a phenomenon is also captured and validated from cavitation-induced vibration and noise effects [16,23]. For other working conditions, the varying trends as shown in figure 12 are also associated with the corresponding cavitation structures within the model pump.

## 4. Conclusion

The cavitation-induced pressure pulsation characteristics of a low specific speed centrifugal pump are investigated in the present paper. Pressure transducers are mounted on the volute casing to obtain unsteady pressure signals at different cavitation numbers and flow rates. Emphasis is placed on the component at the blade-passing frequency *f*_BPF_ and RMS* values in the 0–500 Hz frequency band to clarify and discuss the influence of cavitation development on the pressure spectrum. The main conclusions are as follows.

For points P1–P7, significant peaks at *f*_BPF_ and its harmonic 2*f*_BPF_ can be captured in the pressure spectrum, and the pressure amplitude reaches its maximum at *f*_BPF_. In the cavitation condition, the pressure spectrum characteristics are consistent with those in the non-cavitation condition, and the component at *f*_BPF_ always dominates the pressure spectrum.

For point In1 near the cavitation region, pressure spectra both for amplitudes at *f*_BPF_ and for RMS* values are more easily affected by the cavitation structure. The pressure energy first remains unchanged and then decreases rapidly in a particular cavitation number range, and the amplitude is much smaller than the initial amplitude in the non-cavitation condition. Finally, the pressure energy rises with cavitation numbers lower than the critical point. For points P1–P7, at high cavitation numbers, the pressure energy changes slightly, and it also shows a rapid increase after the critical point.

The decrease in pressure energy in a particular cavitation number range is associated with the corresponding cavitation structure. When the cavitation structure changes from a compressible to incompressible status, the pressure energy experiences decreasing and increasing trends versus the cavitation number.

## Data accessibility

This article does not contain any additional data.
